# Therapeutic Prospect of New Probiotics in Neurodegenerative Diseases

**DOI:** 10.3390/microorganisms11061527

**Published:** 2023-06-08

**Authors:** Mingxia Bi, Chang Liu, Yulin Wang, Shuang-Jiang Liu

**Affiliations:** 1 State Key Laboratory of Microbial Biotechnology, Shandong University, Qingdao 266237, China; bimx@sdu.edu.cn (M.B.);; 2 State Key Laboratory of Microbial Resources and Environmental Microbiology Research Center, Institute of Microbiology, Chinese Academy of Sciences, Beijing 100101, China

**Keywords:** gut microbiome, neurodegenerative diseases, probiotics, *Clostridium butyricum*, *Akkermansia muciniphila*, *Faecalibacterium prausnitzii*, *Bacteroides fragilis*

## Abstract

Increasing clinical and preclinical evidence implicates gut microbiome (GM) dysbiosis as a key susceptibility factor for neurodegenerative disorders, including Alzheimer’s disease (AD) and Parkinson’s disease (PD). In recent years, neurodegenerative diseases have been viewed as being driven not solely by defects in the brain, and the role of GM in modulating central nervous system function via the gut–brain axis has attracted considerable interest. Encouraged by current GM research, the development of new probiotics may lead to tangible impacts on the treatment of neurodegenerative disorders. This review summarizes current understandings of GM composition and characteristics associated with neurodegenerative diseases and research demonstrations of key molecules from the GM that affect neurodegeneration. Furthermore, applications of new probiotics, such as *Clostridium butyricum*, *Akkermansia muciniphila*, *Faecalibacterium prausnitzii*, and *Bacteroides fragilis*, for the remediation of neurodegenerative diseases are discussed.

## 1. Introduction

Age-related neurodegenerative diseases, including Alzheimer’s disease (AD) and Parkinson’s disease (PD), are chronic and progressive neurological disorders with a range of causes and clinical presentations [1,2]. The prevalence of neurodegenerative diseases has increased worldwide in parallel with the rise in life expectancy, and effective treatments for neurodegenerative diseases are highly desired. Of note, the primary prodromal symptom of AD and PD, gastrointestinal dysfunction, is detected prior to clinical diagnosis, suggesting that the gastrointestinal tract and its connection to the central nervous system (CNS) are involved in these diseases’ etiology [3]. Among other causes (genetics, immune system, etc.) attributed to AD and PD pathogenesis [4], gut dysbiosis is an emerging factor and has received increasing attention. The advent of multi-omics sequencing has led to revolutionary advances in our understanding of gut dysbiosis in neurodegenerative diseases [5]. Once it became understood that our gut can effectively communicate with our brain, numerous studies sought to clarify the intricate processes involved. In recent years, a new player has emerged as a key regulator of the gut–brain axis, that is, the gut microbiota (the trillions of microorganisms, including bacteria, fungi, viruses, protists, and archaea, living in the gut). Clinical treatments that target the gut microbiota provide a new and promising approach for reducing risks, modulating symptoms, and delaying neurodegenerative progression. A phenomenon now referred to as pharmacomicrobiomics reveals that drugs can influence gut microbiota composition, and the gut microbiota can also influence an individual’s response to a specific drug.

In this communication, we review recent progress in the understanding of gut dysbiosis and neurodegeneration, with particular emphasis on the potential application of new probiotics for AD and PD therapies.

## 2. Pathological Features of Neurodegenerative Diseases

Neurodegenerative diseases are relatively common and are progressive, and devastating neurological disorders. As the global population ages, the incidence and prevalence of neurodegenerative diseases have risen rapidly in the past two decades. AD is the most prevalent neurodegenerative disease and the leading cause of dementia worldwide. Histopathologically, AD is characterized by β-amyloid (Aβ)-containing extracellular plaques and tau-containing intracellular neurofibrillary tangles [2]. The presentation of AD with memory impairment is most common, but difficulties in expressive speech, visuospatial processing, and executive functions co-occur, ultimately leading to the classic clinical signs of dementia. PD apparently results from the complex interplay of α-synuclein aggregation, neuroinflammation, mitochondrial dysfunction, and abnormal synaptic transmission, leading to the gradual, irreversible loss of dopaminergic neurons in the substantia nigra and the resulting striatal dopamine depletion [6]. In addition to the cardinal motor symptoms, such as resting tremor, rigidity, and bradykinesia, PD patients also exhibit non-motor symptoms, including hyposmia, sleep disorders (e.g., rapid eye movement sleep behavior disorder), psychiatric symptoms (anxiety and depression), cognitive impairment, and gastrointestinal disturbances (constipation, delayed gastric emptying, dysphagia, and sialorrhoea) [7].

In general, Aβ peptides begin to accumulate approximately 10–30 years before the onset of dementia, which occurs in the early stages of AD and is detectable in the basal temporal and medial frontal regions [8]. The early stage of PD is difficult to recognize, and by the time that patients notice their motor symptoms, the disease has usually advanced for a long period. It has been shown that non-motor symptoms, particularly gastrointestinal dysfunction, frequently occur approximately 20 years before neurodegeneration appears. Considering the high prevalence of gastrointestinal symptoms, these symptoms are considered a prodromal phase of neurodegenerative diseases [7]. Specifically, the pathology in the gastrointestinal tract shows similarity with the brain. Aβ and α-synuclein can spread gradually from the enteric nervous system (ENS) to specific brain regions, subsequently causing neurodegeneration [9,10]. More studies are needed to understand the relationship between gastrointestinal symptoms and disease progression, with the aim of discovering new biomarkers for diagnosis. Furthermore, in light of recent developments in gastrointestinal dysfunction, the gut might be a gateway for the development of an urgently needed disease-modifying therapy.

## 3. Gut Microbiome Alterations in Neurodegenerative Diseases

A healthy human gut harbors a microbiome of 200–400 species and trillions of microbial cells. This gut microbiome (GM) is dynamic, and the microbial composition and the abundances of species are affected by environment, diet, age, feeding mode, application of antibiotics, etc. [11]. Notably, the GMs of AD and PD patients have been found to display different features than those displayed by healthy GMs, implicating their role in the pathogenesis of neurodegeneration via the regulation of gut barrier integrity, neuroinflammation, immune responses, and neurotransmitter activities [12,13,14]. GM alterations are mainly in the *Firmicutes* phylum (including the *Oscillospiraceae* family, *Enterococcaceae* family, *Streptococcaceae* family, and *Lachnospiraceae* family) and *Bacteroidetes* phylum (including the *Rikenellaceae* family and *Prevotellaceae* family). At the genus level, fecal samples from AD patients have been found to show increased abundances of *Ruminococcus* [14], *Enterococcus* [15], *Streptococcus* [16], *Alistipes* [17], *Dorea* [16], *Collinsella* [14,15], and *Eggerthella* [15], while there were decreased abundances of *Faecalibacterium* [15], *Lachnospira* [14,18], *Roseburia* [15], and *Coprococcus* [15]. In addition to AD, increasing evidence shows that GM dysbiosis is implicated in PD-related pathology. In many studies, the GMs of PD patients showed alterations at the genus level compared with healthy controls, including increased abundances of *Alistipes* [19], *Streptococcus* [20], *Ruminococcus* [21,22], *Enterobacter* [20,23], *Enterococcus* [20,24], *Verrucomicrobium* [21,25], *Desulfovibrio* [26], and *Anaetroncus* [27], whereas decreases were seen for *Faecalibacterium* [20,25,28], *Prevotella* [12,21,23,29], *Blautia* [20,25,30], *Lachnospira* [25,26], and *Roseburia* [12,25,30]. In summary, current studies have indicated that AD and PD share common GM dynamics, i.e., increased abundances of *Streptococcus*, *Ruminococcus*, and *Alistipes*, and decreased abundances of *Faecalibacterium*, *Lachnospira*, and *Roseburia* (Table 1). Multiple sclerosis (MS) is a chronic inflammatory and degenerative disease of the CNS. Abundant evidence indicates that the GM plays a role in MS through its influence on immune function [31]. In both progressive and relapsing-remitting MS, the abundances of *Clostridium bolteae* and *Ruthenibacterium lactatiformans*, as well as the genera of *Akkermansia* and *Methanobrevibacter*, have been found to be increased, whereas the abundances of *Blautia wexlerae* and *Dorea formicigenerans* have been found to have decreased [32,33]. Furthermore, MS patients have been shown to be more likely to harbor and show an increase in the epsilon toxin-producing strains of *Clostridium perfringens* [34].

GM alterations are also implicated in the clinical manifestations of neurodegenerative diseases. It has been reported that *Dorea*, the *Oscillospira* family, and *Ruminococcus* are positively associated with constipation, whereas *Faecalibacterium* is negatively associated with constipation [35]. Decreased abundances of *Faecalibacterium* and *Roseburia* are correlated with gastrointestinal comorbidities, especially constipation severity [36]. Infection with *Citrobacter rodentium* can induce anxiety-like symptoms that are likely mediated via vagal sensory neurons [37]. Lower levels of *Lachnospiraceae* and higher levels of *Enterobacteriaceae* families are linked with increased disease severity in PD [38]. The gut microbiota of patients with neurodegenerative diseases is significantly altered and exhibits obvious characteristics, which may be applied for the development of potential biomarkers or therapeutic targets. A lack of understanding of the mechanisms and pathophysiology of GM disturbance hampers the diagnosis and clinical treatment of neurodegenerative diseases. Owing to differences in sequencing techniques, diet, regional disparity, and therapeutic regimen, types of altered gut microbiota have not been clearly elucidated. Rigorous and standardized methodology is needed to draw stronger conclusions regarding the question as to whether there exist common microbiome signatures for neurodegenerative diseases. While an increasing number of studies have revealed the association of GM dysbiosis with neurodegenerative diseases, further causative studies are still needed to reveal the mechanisms underlying these diseases and their potential relevance to clinical manifestations.

## 4. Microbiota–Gut–Brain Axis

It is well accepted that the GM exerts considerable influence on brain function via the microbiota–gut–brain axis. The GM communicates with the brain via the activation of the vagus nerve, stimulation of enterochromaffin cells and immune system, and direct transport of metabolites from the circulation into the brain [39]. In regard to the neuronal pathways for gut–brain connections, the vagus nerve is the most direct and well-studied pathway. In mice with autism spectrum disorder (ASD), *Lactobacillus reuteri* was reported to rescue social dysfunction in a vagus nerve-dependent manner [40]. It has been found that microbial production of indole from tryptophan was more likely to result in anxiety and depression in the host because bacterial indole could activate vagal neurons and negatively impact emotional behaviors [41]. The propagation of Aβ and α-synuclein in the gastrointestinal tract has been found to be transmitted via the vagus nerve to the brain [42,43]. Colonic enterochromaffin cells express receptors for various GM-derived metabolites, such as short-chain fatty acids (SCFAs), aromatic amino acids, secondary bile acids, and neurotransmitters [44,45]. Furthermore, enterochromaffin cell production of serotonin has the potential to influence brain function directly or indirectly [46]. For immune-mediated routes, SCFAs interact closely with the immune system through the activation of G protein-coupled receptor (GPCR) and inhibition of histone deacetylase (HDAC) activity, leading to decreased neuroinflammation [47,48]. Ghrelin, a brain–gut peptide mainly released by X/A-like cells of the stomach, has been shown to elicit neuroprotective effects in both AD and PD [49,50]. Specific gastrointestinal microbiota and their metabolites are able to modulate ghrelin secretion. Microbial-derived SCFAs and hydrogen sulfide regulate circulating ghrelin levels via direct or indirect modulation of ghrelin secretion [51]. Hydrogen supplementation can also increase the concentration of ghrelin, and the neuroprotective effects of hydrogen can be abolished by ghrelin receptor antagonists [52]. On the other hand, a bacterial endotoxin, lipopolysaccharide (LPS), has been shown to aggravate neuroinflammation by directly entering the brain or by activating immune response [53].

## 5. Linking Gut Microbiome Dysbiosis and Neurodegenerative Diseases

### 5.1. Gut Microbiome Interacts with Hosts Subsection

**Figure 1:**
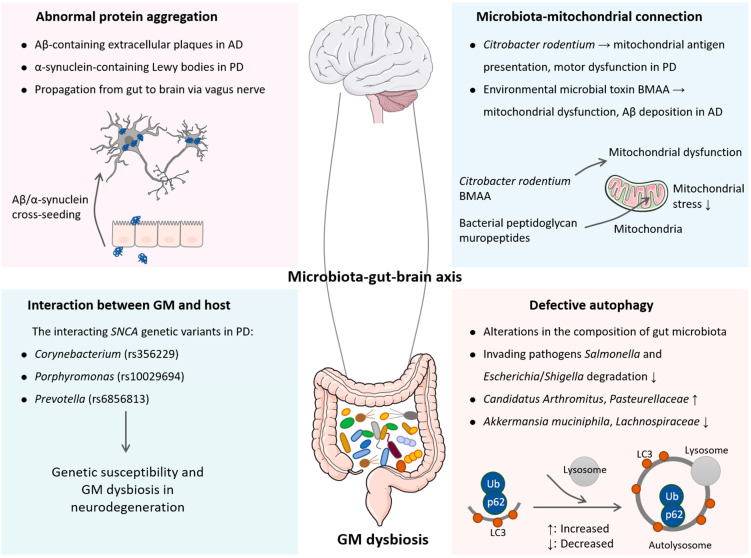
Potential mechanisms of gut microbiome dysbiosis involved in neurodegenerative diseases. AD is characterized by Aβ-containing extracellular plaques, and PD is characterized by intracellular α-synuclein accumulation to form Lewy bodies. The propagation of Aβ and α-synuclein in the gastrointestinal tract can be transmitted via the vagus nerve to the brain. In addition, there is an interaction between host genetic susceptibility to neurodegeneration and GM dysbiosis, that is, *Corynebacterium*, *Porphyromonas*, and *Prevotella* interact with the *SNCA* genetic variants rs356229, rs10029694, and rs6856813, respectively. *Citrobacter rodentium* and environmental microbial neurotoxin BMAA trigger mitochondrial dysfunction, ultimately leading to neurodegeneration. Furthermore, defective autophagy fails to eliminate intracellular pathogens and induces alterations in the composition of the gut microbiome.

The GM–host interaction is an important direction to understand the regulation of health and disease. As previously reported, the GMs of patients with bipolar disorder depression were sufficient to induce depression-like behavior in mice, which was attributed to the elevated expression of tetratricopeptide repeat and ankyrin repeat containing 1 *(TRANK1)*, a robust risk gene of bipolar disorder [54]. Inspiringly, the interplay between the GM and its host has been investigated in PD. Intracellular protein aggregates that are primarily composed of α-synuclein in Lewy bodies serve as the neuropathological hallmark of PD. α-Synuclein is encoded by the *SNCA* gene, the mutations of which lead to a drastic overexpression of α-synuclein and cause a Mendelian autosomal-dominant form of PD [55]. Given that there is an overabundance of opportunistic pathogens in PD, the question as to whether these pathogens are triggers of the neurodegeneration seen is being investigated, and there is likely a connection to *SNCA* variants. Recently, Wallen et al. [56] have reported the association of three opportunistic pathogens with PD, which is dependent on *SNCA* genetic variations. The candidate *SNCA* genetic variants for interaction with the genera *Corynebacterium*, *Porphyromonas*, and *Prevotella* are rs356229, rs10029694, and rs6856813, respectively. Among these, the *Porphyromonas* interacting genetic variant is also associated with increased PD risk. These findings indicate that the increased abundance of opportunistic pathogens seen in the PD gut might be modulated by host genotype. In this sense, there is an interaction between genetic susceptibility to the disease and GM dysbiosis (Figure 1). Nonetheless, the power of a single gene in explaining the interaction between a host and GM is limited, and the conclusions may be partial and misleading [57]. Further studies to integrate multiple genetic variations in experimental models and humans will be needed to tease out these interactions.

### 5.2. Gut Microbiome–Mitochondria Connection

As the endosymbiosis hypothesis has demonstrated, mitochondria are descendants of primordial aerobic pleomorphic bacteria (likely *Rickettsia*), which developed a mutualistic partnership with ancient anaerobic microbes (likely *Archaea*) [58]. As a consequence, a stable symbiosis was established to provide energy for the host. Bacterial peptidoglycan muropeptides, a unique component of bacterial cell walls in both Gram-positive and Gram-negative species, accumulate in host intestinal mitochondria, which can maintain mitochondrial homeostasis and suppress host mitochondrial oxidative stress [59]. A previous study has shown that intestinal infection with the Gram-negative bacterium *Citrobacter rodentium* can trigger mitochondrial antigen presentation. Subsequently, cytotoxic mitochondria-specific CD8^+^ T cells in *Pink1*-knockout mice deplete dopamine-producing neurons through autoimmune attack, thereby causing the transient motor dysfunction resembling that is seen in PD patients (Figure 1) [60]. β-N-methylamino-L-alanine (BMAA), a natural neurotoxin produced by cyanobacteria or other microbes, has been shown to be involved in neurodegeneration. In a previous study, BMAA was detected in the brains of patients with neurodegenerative diseases, which can cross the blood–brain barrier (BBB) [61]. Mechanistically, BMAA elicits mitochondrial dysfunction and AD features in cortical neurons with increased tau phosphorylation and Aβ peptide deposition [62]. Recently, Esteves et al. [63] reported that BMAA triggered a chain of events including mitochondrial dysfunction and innate immunity activation. When BMAA reaches the gut, it can interact with the ENS and possibly target mitochondria, which has been advanced as a potential cause for neurodegeneration [64].

### 5.3. Defective Autophagy

Autophagy, in a broad sense, refers to a cellular homeostatic mechanism delivering cytoplasmic constituents to lysosomes for degradation. Initially described as a “self-eating” survival pathway that enables nutrient recycling during starvation, autophagy can also respond to a range of inputs, including microbial products commonly known as pathogen-associated molecular patterns (PAMPs) [65]. One of the best appreciated manifestations of autophagy is to defend against microbial invasion through direct elimination of intracellular pathogens [66]. Autophagy degrades invading pathogens (e.g., *Salmonella* and *Escherichia*/*Shigella*), modulates the release of proinflammatory cytokines, and participates in antigen presentation. In intestinal epithelial cells, autophagy enhances the tight junction barrier function owing to the reduced permeability of ions and small molecules due to lysosomal degradation of claudin-2 [67]. Furthermore, autophagy in colonic epithelial cells has been reported to protect against colitis through the maintenance of antimicrobial peptides and secretion of mucins that act as a mucosal barrier against bacterial invasion [68,69]. The disruption of autophagy in intestinal epithelial cells induces alterations in the composition of gut microbiota and reduces α-diversity. In autophagy-deficient mice, the abundances of *Candidatus Arthromitus* and *Pasteurellaceae* family are increased, whereas the abundances of the *Akkermansia muciniphila* and *Lachnospiraceae* families are found to be reduced (Figure 1) [69]. Indeed, both AD and PD are accompanied by defective autophagy, leading to the failure in eliminating protein aggregates or damaged mitochondria [70,71]. Given the impact of autophagy dysfunction in gastrointestinal homeostasis, there is therapeutic interest in activating autophagy to eliminate pathogenic bacteria and protein aggregates, and thus halting the progression of neurodegenerative diseases [72].

## 6. New Probiotics in Neurodegenerative Diseases

According to the Food and Agriculture Association (FAO), probiotics are “live microorganisms, which when administered in adequate amounts confer a health benefit to the host” [73,74]. It has been shown that *Lactobacillus plantarum* PS128 alleviates nigral dopaminergic neuronal death and motor deficits in a PD mouse model [75]. *L. plantarum* DR7 can reduce the dopamine metabolism-related enzymes, β-hydroxylase and tyrosine hydroxylase, to regulate dopamine pathways [76]. *Lactobacillus acidophilus* EG004 shows a positive effect on cognitive ability in a healthy mouse model, probably by producing butyrate and, therefore, modulating neurotransmitters and neurotrophic factors [77]. The administration of *Lacticaseibacillus rhamnosus* Fmb14 has been reported to improve colitis-related depression-like behavior [78]. Human *Lactobacillus brevis* and *Bifidobacterium dentium* are also efficient GABA producers, and have the potential to improve depression-like abnormalities [79,80]. Combined administration of *L. plantarum* and *Bifidobacterium bifidum* with interval aerobic exercise has been found to play a neuroprotective role in AD [81]. Another probiotics mixture (*Lactobacillus rhamnosus*, *Bifidobacterium animalis lactis*, and *L. acidophilus*) can also rescue nigral dopaminergic neurons in PD models by increasing the levels of butyrate [82].

New probiotics are microbial taxa that conform to the traditional definition of probiotics but have not been applied for health improvement. These novel probiotics also contain live organisms, such as bacteria, which can be expected to prevent or treat diseases or improve the health conditions of human beings [73]. Currently, studies investigating new probiotics are ongoing worldwide. Traditional probiotic strains are usually obtained from gut microbiota, milk, and fermented food. Probiotics that are currently available generally belong to a narrow range of microbial species, mainly related to *Lactobacillus* and *Bifidobacterium*. By contrast, new probiotics are isolated from host commensal bacteria using new tools, which harbor a wider range of species and more candidate bacteria. In this paper, we describe several promising probiotics (*Clostridium butyricum*, *Akkermansia muciniphila*, *Faecalibacterium prausnitzii*, and *Bacteroides fragilis)* and their potential applications in neurodegenerative diseases.

### 6.1. Clostridium butyricum

*Clostridium butyricum*, a butyrate-producing, spore-forming anaerobic bacterium, is found in a wide variety of environments, including soil, milk, and vegetables. *C. butyricum* is detected in 10–20% of the human gastrointestinal tract and is one of the earliest colonizers in infants [83]. Traditionally, *C. butyricum* has been used as a potent probiotic owing to its beneficial effects on host health. Because of its increased butyrate production, *C. butyricum* is able to enhance the thickness of the mucosal layer and strengthen the gut barrier integrity by increasing the expression of tight junction proteins (e.g., occludin and ZO-1). In addition, *C. butyricum* plays a protective role in gastrointestinal infections and regulates the host immune system [84]. *C. butyricum* is effective against *Clostoridioides difficile*, a causative pathogen of nosocomial infections; *Helicobacter pylori*, a causative pathogen of gastric cancer; and antibiotic-resistant *Escherichia coli, Staphylococcus aureus*, and *Vibrio cholerae* infections [85,86,87]. It has been shown that *C. butyricum* can also upregulate protectin D1, an anti-inflammatory lipid metabolite, in colon tissue under antibiotic therapy to alleviate systemic inflammation [88]. Indigenous spore-forming bacteria, predominantly *Clostridia*, can promote the biosynthesis of serotonin [44].

**Figure 2:**
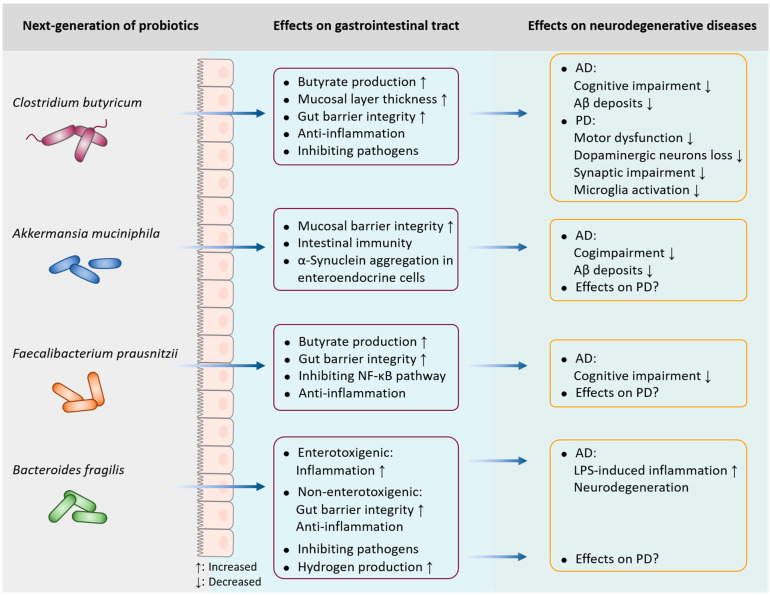
Implications of new probiotics in neurodegenerative diseases. *Clostridium butyricum*, a butyrate-producing anaerobic bacterium, plays a protective role in neurodegenerative diseases; it can prevent cognitive impairment and Aβ deposits in AD, and improve motor deficits, dopaminergic neuron loss, synaptic dysfunction, and microglial activation in PD. *Akkermansia muciniphila* and *Faecalibacterium prausnitzii* are effective in alleviating cognitive deficits and reducing Aβ levels in AD; however, their potential role in PD is unclear. Additionally, enterotoxigenic *Bacteroides fragilis* drives LPS-induced inflammation and degenerative neuropathology in AD, while non-enterotoxigenic *B. fragilis* exerts anti-inflammatory properties. Although *B. fragilis* accounts for the level of intestinal hydrogen in PD, its exact role in the neurodegeneration of PD has not yet been elucidated.

*C. butyricum* exerts neuroprotective effects in various neurodegenerative diseases. In a PD mouse model, the oral administration of *C. butyricum* can improve motor deficits, dopaminergic neuron loss, synaptic dysfunction, and microglial activation. These neuroprotective effects may be related to the increased levels of colonic glucagon-like peptide-1 (GLP-1) and cerebral GLP-1 receptor, eventually restoring gut microbiota homeostasis [89]. Moreover, the anti-depressive effects of *C. butyricum* in chronic, unpredictable and mild stress-induced depression-like behavior may result from the stimulation of intestinal GLP-1 secretion [90]. In AD models, the administration of *C. butyricum* for four weeks prevents cognitive impairment, Aβ deposits, and neuroinflammation, which are mediated by the restoration of gut microbiota and butyrate production (Figure 2) [91]. In vascular dementia mice, *C. butyricum* significantly alleviates the cognitive dysfunction and histopathological changes via anti-apoptotic properties and subsequent activation of the PI3K/Akt pathway [92]. Treatment with *C. butyricum* defends against cerebral ischemia/reperfusion injury through antioxidant and anti-apoptotic mechanisms, which may be partially attributed to the increased butyrate contents in the brain [93]. *C. butyricum* treatment has been consistently shown to improve neurological dysfunction and neurodegeneration in a mouse model of traumatic brain injury [94]. Although the neuroprotective effects of probiotic *C. butyricum* appear well established, additional human randomized controlled trials would further provide valuable clinical data related to various strains’ utility as an intervention in neurodegenerative diseases.

### 6.2. Akkermansia muciniphila

*Akkermansia muciniphila*, a Gram-negative, anaerobic bacterium first identified in 2004 [95], is considered a promising candidate of new probiotics [96]. The benefits of *A. muciniphila* are not limited to protecting the mucosal barrier integrity and improving host metabolic functions and immune responses; *A. muciniphila* also possesses therapeutic value in modulating brain function. In this case, the critical role of *A. muciniphila* has been demonstrated in neurodegenerative diseases. Several studies have consistently reported that the genus *Akkermansia* is highly effective in distinguishing PD or in serving as a potential early biomarker for PD diagnosis [25,29,97]. It is noteworthy that the abundance of *A. muciniphila* is also found to be increased in PD patients [98]. One of the possible explanations for this is that the increased abundance of *A. muciniphila* in PD patients is aimed at fighting and preventing disease progression. Although treatment with *A. muciniphila* has been reported to improve cognitive deficits and reduce Aβ levels in an AD mouse model [99], whether *A. muciniphila* can alleviate neurodegeneration in PD patients remains unknown (Figure 2). There are also opposite results demonstrating that an *A. muciniphila*-conditioned medium can initiate α-synuclein aggregation in enteroendocrine cells [100]. To date, few studies have explored the direct impact of *A. muciniphila* on the nervous system; thus, the likelihood of beneficial effects exerted by *A. muciniphila* should be addressed. Further characterization of its relevance in neurodegeneration will be fundamental to unveil the consequences of *A. muciniphila* dysbiosis.

### 6.3. Faecalibacterium prausnitzii

*Faecalibacterium prausnitzii*, an anaerobic Gram-positive bacterium, belongs to the *Firmicutes* phylum and the *Ruminococcaceae* family, also known as *Clostridium* cluster IV. The interest in *F. prausnitzii* is related to its capacity to produce beneficial metabolites, such as fructose, formic acid, and d-lactate, and it is one of the most important butyrate-producing bacteria [101]. In addition, a 15 kDa protein with anti-inflammatory properties produced by *F. prausnitzii* can inhibit the nuclear factor-κB (NF-κB) pathway in intestinal epithelial cells and prevent colitis in animal models [102]. Consistently, several other studies in mice have clarified the protective role of *F. prausnitzii* in experimentally induced colitis. Intragastric administration of either *F. prausnitzii* or its culture supernatant can significantly decrease the severity of colitis by down-regulating pro-inflammatory cytokines [103]. Butyrate produced by *F. prausnitzii* modulates Th17/Treg balance and exerts anti-inflammatory effects in a colorectal colitis rat model [104]. In terms of neurodegenerative diseases, the abundance of *F. prausnitzii* has been found to be decreased in a group with mild cognitive impairment (MCI) compared with the healthy controls, which correlated with cognitive scores [105]. Two isolated *F. prausnitzii* strains from healthy individuals have been shown to improve cognitive impairment in an AD mouse model [105]. Thus far, studies on the potential effects of *F. prausnitzii* on PD have not been reported (Figure 2). Additional research studies are needed to further prove the beneficial role of *F. prausnitzii* in the remediation of neurodegenerative diseases. 

### 6.4. Bacteroides fragilis

*Bacteroides fragilis* is another promising probiotic, and is a commensal, Gram negative, non-spore-forming obligatory anaerobic bacterium abundant in the human gastrointestinal tract. Typically, *B. fragilis* can interfere with other microbes by inhibiting their growth or translocation. As previously reported, *B. fragilis* treatment prevents *Clostrioides difficile* infection, possibly by resisting pathogen colonization, enhancing the relative abundance of *A. muciniphila*, and improving the gut barrier integrity [106]. Indeed, *B. fragilis* can be classified into two subgroups: non-enterotoxigenic and enterotoxigenic *B. fragilis* [107]. Enterotoxin-containing *B. fragilis* secretes an unusually complex mixture of neurotoxins, including pro-inflammatory LPS. In this sense, exposure to enterotoxigenic *B. fragilis* in human primary brain cells is an exceptionally potent inducer of the inflammatory pathway, driving pro-inflammatory degenerative neuropathology in the AD brain [108]. Conversely, non-enterotoxigenic *B. fragilis* strains exert beneficial effects owing to their anti-inflammatory and immunomodulatory activities. The oral administration of *B. fragilis* has been found to increase gut microbiota diversity and beneficial commensal bacteria, thereby improving the gut tight junction integrity and reducing inflammatory cytokines [109]. Fecal bacterial assessment based on 16S rRNA amplicon sequencing shows that the abundance of *B. fragilis* is lower in PD patients than in healthy controls [110]. Considering that *B. fragilis* is one of the main hydrogen-producing intestinal bacteria, the decreased abundance of *B. fragilis* may be accounted for by the lower amount of intestinal hydrogen level in PD patients (Figure 2) [111]. Although *B. fragilis* is potentially interesting as a new probiotic, its role in neuropathology is contrasting, thus the specific strains used should be carefully evaluated for their safety and efficacy in neurodegenerative diseases.

## 7. Perspectives and Conclusions

Gastrointestinal dysfunction serves as a prodromal symptom preceding the clinical manifestations of neurodegenerative diseases. Currently, there is growing interest in new probiotics as potential therapeutic agents. The safety and tolerability of these novel probiotics need to be validated in both animal models and human trials in order to develop personalized applications. Understanding the mechanisms by which probiotics colonize in the gut could lead to the development of “personalized” bacterial therapies. Another challenge is storage due to the strict anaerobic conditions required during microbial collection and freeze drying. Even so, the current state of new probiotics remains largely promising in the context of neurodegenerative diseases for the purpose of slowing down or preventing neurodegeneration, as well as developing effective therapeutic interventions.

## Figures and Tables

**Table 1:** Gut microbiome alterations in neurodegenerative diseases.

Subjects	Gut Microbiome Alterations	
	Increased	Decreased
AD patients	*Ruminococcus* [14] *Enterococcus* [15] *Streptococcus* [16] *Alistipes* [17] *Dorea* [16] *Collinsella* [14,15] *Eggerthella* [15]	*Faecalibacterium* [15] *Lachnospira* [14,18] *Roseburia* [15] *Coprococcus* [15]
PD patients	*Alistipes* [19] *Streptococcus* [20] *Ruminococcus* [21,22] *Enterobacter* [20,23] *Enterococcus* [20,24] *Verrucomicrobium* [21,25] *Desulfovibrio* [26] *Anaetroncus* [27]	*Faecalibacterium* [20,25] *Prevotella* [21,23,29] *Blautia* [20,25,30] *Lachnospira* [25,26] *Roseburia* [25,30]
MD patients	*Clostridium bolteae* [32] *Ruthenibacterium lactatiformans* [32] *Clostridium perfringens* [34] *Akkermansia* [32,33] *Methanobrevibacter* [33]	*Blautia wexlerae* [32] *Dorea formicigenerans* [32]

## Data Availability

Not applicable.

## Abbreviations

Aβ: β-amyloid; AD: Alzheimer’s disease; ASD: autism spectrum disorder; BBB: blood–brain barrier; BMAA: β-N-methylamino-L-alanine; CNS: central nervous system; ENS: enteric nervous system; GLP-1: glucagon-like peptide-1; GM: gut microbiome; GPCR: G protein-coupled receptor; HDAC: histone deacetylase; LPS: lipopolysaccharide; MCI: mild cognitive impairment; NF-κB: nuclear factor-κB; PAMP: pathogen-associated molecular pattern; PD: Parkinson’s disease; SCFAs: short-chain fatty acids; *TRANK1*: tetratricopeptide repeat and ankyrin repeat containing 1.
